# Vortex lattice in the crossover of a Bose gas from weak coupling to unitarity

**DOI:** 10.1038/s41598-018-27146-1

**Published:** 2018-06-11

**Authors:** S. K. Adhikari, L. Salasnich

**Affiliations:** 1 Instituto de Física Teórica, UNESP - Universidade Estadual Paulista, 01.140-070 São Paulo, São Paulo, Brazil; 2 Dipartimento di Fisica e Astronomia “Galileo Galilei”, Università di Padova, Via Marzolo 8, 35131 Padova, Italy; 3 Istituto Nazionale di Ottica (INO) del Consiglio Nazionale delle Ricerche (CNR), Via Nello Carrara 1, 50019 Sesto Fiorentino, Italy

## Abstract

The formation of a regular lattice of quantized vortices in a fluid under rotation is a smoking-gun signature of its superfluid nature. Here we study the vortex lattice in a dilute superfluid gas of bosonic atoms at zero temperature along the crossover from the weak-coupling regime, where the inter-atomic scattering length is very small compared to the average distance between atoms, to the unitarity regime, where the inter-atomic scattering length diverges. This study is based on high-performance numerical simulations of the time-dependent nonlinear Schrödinger equation for the superfluid order parameter in three spatial dimensions, using a realistic analytical expression for the bulk equation of state of the system along the crossover from weak-coupling to unitarity. This equation of state has the correct weak-coupling and unitarity limits and faithfully reproduces the results of an accurate multi-orbital microscopic calculation. Our numerical predictions of the number of vortices and root-mean-square sizes are important benchmarks for future experiments.

## Introduction

At ultralow temperatures a dilute gas of bosonic atoms undergoes a phase transition to a superfluid state of matter known as a Bose-Einstein condensate. Soon after the observation of Bose-Einstein condensates in alkali-metal atoms^1–3^, experiments revealed^4^ the formation of vortices in the form of triangular lattice in a rapidly rotating Bose-Einstein condensate demonstrating its superfluid nature. For such dilute quantum gases the effective range of the inter-atomic interaction is much smaller than the average distance between two atoms. Under this condition the interaction can be characterized by a single parameter, the so-called s-wave atomic scattering length *a*^5,6^ taken to be positive (repulsive interaction) throughout this study. Most experiments on Bose-Einstein condensates were performed in the weak-coupling limit characterized by small values of the gas parameter *x* ≡ *n*^1/3^*a* ($x\ll 1$)^6^, where *n* is the density of the gas. The theoretical description of such a system is based on a mean-field nonlinear equation, known as the Gross-Pitaevskii equation^7,8^, where the nonlinearity is determined by^6^ the bulk chemical potential of a uniform gas written as a function of the density *n* and the scattering length *a*.

Quite remarkably, the scattering length *a* can now be routinely manipulated by varying an external magnetic field near a Feshbach resonance, thus changing effectively the inter-atomic interaction^9,10^. As the scattering length *a* becomes much larger than all length scales of the system in the strong-coupling regime characterized by large values of the gas parameter ($x\gg 1$) the system exhibits universal behavior^11–17^ determined only by the density *n* of the gas. The unitary limit, where *x* → +∞, can be achieved by increasing the scattering length to divergent values near a Feshbach resonance and is characterized by simple universal laws arising from scale invariance. This limit is of great interest in fields as diverse as ultra-cold atoms^13–17^, quark-gluon plasma^18^, neutron^19^ and Bose^20–23^ stars, superconductivity^24^, and string theory^25^. The unitary Fermi gas made of fermionic alkali-metal atoms has been largely investigated^26^ both experimentally and theoretically. In the case of bosonic atoms there are technical difficulties associated with a large three-body loss by molecule formation as the unitarity limit is approached by increasing the scattering length *a*^27,28^. Only recently these difficulties have been overcome to observe a unitary Bose gas^15^.

In the commonly studied weak-coupling limit ($x\ll 1$), the zero-temperature bulk chemical potential *μ*(*n*, *a*) of a uniform three-dimensional (3D) Bose gas is given by

1 $$\mu (n,a)=4\pi na+2\pi \alpha {n}^{3/2}{a}^{5/2}+\cdots ,\,\alpha =\frac{64}{3\sqrt{\pi }},$$

where the first term is the familiar mean-field result, while the second term is the beyond-mean-field Lee-Huang-Yang correction^29^ which becomes important as *a* increases. Equation (1) is the zero-temperature equation of state for a weakly-interacting Bose gas, containing the mean-field contribution and also the first beyond-mean-field correction (for a recent review see^30^). Here we use *ħ* = *m* = 1 with *ħ* the reduced Planck constant and *m* the mass of a boson. By dimensional arguments, the bulk chemical potential at unitarity is instead proportional to *n*^2/3^, namely^15,27,28^

2 $$\mathop{\mathrm{lim}}\limits_{a\to \infty }\,\mu (n,a)=\eta \,{n}^{2/3},$$

where *η* is a universal parameter. Although there is no experimental estimate of the parameter *η* for bosons, there are some recent many-body calculations. Ding and Greene (DG)^31^ performed a microscopic multi-orbital calculation of *μ*(*n*, *a*) along the crossover and obtained *η* = 4.7. Also other theoretical predictions for *η*^32–36^ lie in the range from 3 to 9, except *η* = 22.22 of ref.^37^. In this study we suggest an analytic expression for the zero-temperature bulk chemical potential *μ*(*n*, *a*), which reduces to Eqs (1) and (2) in the appropriate limits and which faithfully reproduces the results of the recent multi-orbital calculation by Ding and Greene^31^.

From Eqs (1) and (2), we assume that the zero-temperature bulk chemical potential *μ*(*n*, *a*) along crossover from weak coupling to unitarity can be written as

3 $$\mu (n,a)={n}^{2/3}f(x),\,x\equiv {n}^{1/3}a,$$

where *f*(*x*) is a dimensionless universal function. In this paper we propose a *parameter-free minimal analytical* expression for the crossover function *f*(*x*), incorporating the weak-coupling regime, Eq. (1), and the the unitarity limit, Eq. (2), given by

4 $$f(x)=4\pi \frac{x+\alpha {x}^{5/2}}{1+\frac{\alpha }{2}{x}^{3/2}+\frac{4\pi \alpha }{\eta }{x}^{5/2}}.$$

The presence of quantized topological configurations, like vortices, is a clear signature of the existence of an underlying superfluid order parameter, which plays the key role in all superfluid to normal phase transitions^5,38^. Quantized vortices have been found in superconductors^39^, superfluid helium^40^ and in trapped degenerate gas^4,41,42^. A vortex-lattice structure has been created in a rapidly rotating dilute trapped Bose-Einstein condensate. Here we study the vortex-lattice generation in a rotating disk-shaped trapped bosonic superfluid along the weak coupling to unitarity crossover, performing open-multi-processor-parallelized numerical simulations^43,44^ of the nonlinear Schrödinger equation of the superfluid order parameter^45^, whose nonlinear term is proportional to the chemical potential of Eqs (3) and (4). Throughout this study, we will assume the system to be an ideal superuid at zero Kelvin temperature. Not surprisingly, comparing these results for vortex lattice with those obtained from the simple mean-field Gross-Pitaevskii equation^7,8^, i.e. by using

5 $$\mu (n,a)=4\pi na,$$

we find that the latter may lead to a qualitatively incorrect description. See^46–55^ for previous numerical results with the Gross-Pitaevskii equation. The properties of a rotating dilute bosonic superfluid and of the generated vortex lattice, and in particular the number of vortices and root-mean-square (rms) sizes, are very sensitive to the value of the scattering length through the bulk chemical potential *μ*(*n*, *a*). In other words, different forms of *μ*(*n*, *a*) lead to widely different vortex-lattice structures. Our numerical simulations can be quite useful for future experiments on the formation of quantized vortices in bosonic systems made of alkali-metal atoms.

## Results

### Superfluid hydrodynamics and nonlinear equations

#### General Consideration

According to Tisza^56^ and Landau^57^, at zero temperature the equations of superfluid hydrodynamics for a dilute degenerate Bose gas are given by^5,6^

6 $$\frac{\partial n}{\partial t}+{\boldsymbol{\nabla }}\cdot (nv)=0,$$

7 $$\frac{\partial {\bf{v}}}{\partial t}+{\boldsymbol{\nabla }}[\frac{1}{2}{v}^{2}+V({\bf{r}})-\frac{{\nabla }^{2}\sqrt{n}}{2\sqrt{n}}+\mu (n,a)]=0,$$

where *n*(**r**, *t*) is the superfluid density, **v**(**r**, *t*) the superfluid velocity, and *V*(**r**) is the external trapping potential. These equations of a nonviscous, irrotational fluid describe accurately many collective properties of a bosonic superfluid^5,38^. The quantum pressure term $-\,{\nabla }^{2}\sqrt{n}/(2\sqrt{n)}$, which is absent in the original equations of Tisza^56^ and Landau^57^, is indeed necessary to model surface effects^6^. It is important to stress that, in general, at zero temperature a bosonic system is fully superfluid also if its condensate fraction can be small. This is the case of ^4^He liquid (where the effective range of the inter-atomic potential is of the order of the interparticle distance), whose zero-temperature condensate fraction is less than 10%^5^. In the case our dilute bosonic gas, characterized by an effective range of the interaction much smaller than the interparticle distance, the zero-temperature condensate fraction is close to 100% in the weak-couling regime and it reduces to about 80% at unitarity^36^.

As suggested by Onsager^58^, Feynman^59^ and Abrikosov^60^, superfluids have another amazing property: the circulation of the superfluid velocity field **v**(**r**, *t*) around a generic closed path ${\mathscr{C}}$ is quantized, namely^61^

8 $${\oint }_{C}\,{\bf{v}}\cdot d{\bf{r}}=2\pi \,q,$$

where *q* = 0, ±1, ±2, …. is an integer number. If *q* ≠ 0 it means that inside the closed path ${\mathscr{C}}$ there are topological defects, and the domain where **v** is well defined is multiply connected. A simple example of topological defect is a quantized vortex line. Nowadays quantized vortices are observed experimentally in type-II superconductors^62,63^, in superfluid liquid helium^64^, and in ultra-cold atomic gases^5,38,61^. The quantization of circulation can be explained following the old intuition of London^65^ and assuming that the dynamics of superfluids is driven by a complex scalar field (for an in-depth discussion see^38,61^)

9 $$\varphi ({\bf{r}},t)=|\varphi ({\bf{r}},t)|{e}^{i\theta ({\bf{r}},t)},$$

which satisfies the nonlinear Schrödinger equation^6^

10 $$i\frac{\partial }{\partial t}\varphi ({\bf{r}},t)=[-\frac{1}{2}{\nabla }^{2}+V({\bf{r}})]\,\varphi ({\bf{r}},t)+\mu (n,a)\,\varphi ({\bf{r}},t)$$

with $n({\bf{r}},t)=N|\varphi ({\bf{r}},t){|}^{2},\,\int \,|\varphi ({\bf{r}},t){|}^{2}d{\bf{r}}=1$ and the phase *θ*(**r**, *t*) defines the superfluid velocity ${\bf{v}}({\bf{r}},t)={\boldsymbol{\nabla }}\theta ({\bf{r}},t)$, where *N* is the number of atoms.

In fact, under these assumptions, Eq. (10) is equivalent to Eqs (6) and (7) and the multi-valued angle variable *θ*(**r**, *t*) gives rise to Eq. (8). The complex field *ϕ*(**r**, *t*) is the superfluid order parameter of our zero-temperature theory. In other words, *ϕ*(**r**, *t*) is the wavefunction of our single-orbital theory for a system that is fully superfluid. Notice that for *μ*(*n*, *a*) = 4*πna*, i.e. for a very weakly interacting bosonic superfluid, the nonlinear Schrödinger equation reduces to the familiar Gross-Pitaevskii equation^7,8^ characterized by a cubic nonlinearity. Remarkably, only very recently, vortex arrays in Fermi superfluids through the crossover from the weak-coupling situation with largely overlapping atomic pairs to the strong-coupling limit where composite bosons form and condense, have been studied numerically by using a differential equation for the local order parameter, obtained by coarse graining the Bogoliubov-de Gennes equations^66^.

#### Bulk chemical potential

The bulk chemical potential of Eq. (3) includes the effect of atomic interaction on the properties of the trapped dilute bosonic superfluid governed by Eq. (10). Different functional forms of *f*(*x*) may lead to widely different properties, specially, of the generated vortex lattice in a rapidly rotating trapped bosonic superfluid. Similar crossover functions have been suggested and used mostly in the case of a Fermi gas^11,12,67,68^.

**Figure 1:**
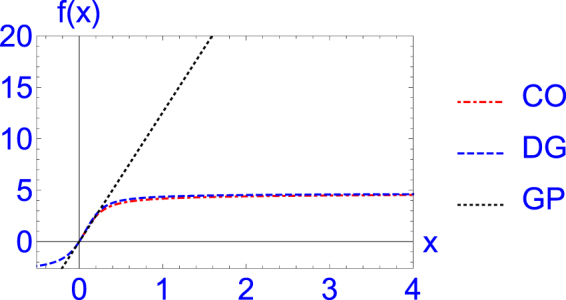
Dimensionless function *f*(*x*) of the zero-temperature bulk chemical potential *μ* = *n*^2/3^*f*(*x*) versus *x* = *n*^1/3^*a*. CO: the crossover function (4) with *η* = 4.7, DG: multi-orbital calculation of Ding and Greene^31^, GP: Gross-Pitaevskii function *f*(*x*) = 4*πx*.

In Fig. 1 we display the crossover function (4) for *η* = 4.7, and compare it with that of the Gross-Pitaevskii model, Eq. (5), and the recent microscopic multi-orbital Hartree calculation of Ding and Greene^31^. We find that the crossover function agrees well with the multi-orbital Hartree calculation for all *x*. The Gross-Pitaevskii function, *f*(*x*) = 4*πx* is reliable only for a relatively small value of the gas parameter *x*.

#### Rapidly rotating degenerate Bose gas

Abrikosov^60^ demonstrated from energetic consideration that a rapidly rotating Bose gas prefers many vortices of unit angular momentum per atom arranged in a regular lattice over a vortex of multiple angular momentum. It has been demonstrated^46–53^ that the formation of such vortex lattice in a trapped degenerate Bose gas can be well described by the following stationary dimensionless equation in the rotating frame obtained upon the inclusion of a term −Ω*L*_*z*_ in Eq. (10) ^69^:

11 $$\begin{array}{rcl}i\frac{\partial \varphi ({\bf{r}},t)}{\partial t} & = & [-\frac{1}{2}{\nabla }_{{\bf{r}}}^{2}+\mu (n,a)+i{\rm{\Omega }}(x\frac{\partial }{\partial y}-y\frac{\partial }{\partial x})\\  &  & +\frac{1}{2}({\gamma }^{2}{x}^{2}+{\nu }^{2}{y}^{2}+{\lambda }^{2}{z}^{2})]\,\varphi ({\bf{r}},t),\end{array}$$

where Ω is the frequency of rotation about *z* axis and *L*_*z*_ is the *z* component of angular momentum: $${L}_{z}\equiv $$$$-\,i\hslash (x\frac{\partial }{\partial y}-y\frac{\partial }{\partial x})$$. In Eq. (11) the anisotropies *γ*, *ν*, *δ* of the 3D trap *V*(**r**) along *x*, *y*, *z* axes, respectively, are explicitly shown in units of an overall frequency *ω* of the confining trap, the length is expressed in units of $$l\equiv \sqrt{\hslash /m\omega }$$, the angular frequency Ω is expressed in units of *ω*, time *t* in units of *ω*^−1^, *μ* in units of *ħω*, *ϕ* in units of *l*^−3/2^. The stable vortex lattice emerges as the ground state of Eq. (11), which we study numerically by imaginary-time propagation^52,53^.

#### Dimensional reduction

Many experiments are performed in strongly disk- or cigar-shaped condensates. The present crossover model leads to useful quasi two-dimensional (2D) or one-dimensional (1D) nonlinear models in these cases, which are included here for the sake of completeness. If we have a stronger trap in the *z* direction ($\lambda \gg \nu ,\gamma$), the quasi-2D nonlinear Schrödinger equation for the weak-coupling to unitarity crossover can be written as (viz. Methods for details):

12 $$\begin{array}{rcl}i\frac{\partial {\varphi }_{2D}(x,y,t)}{\partial t} & = & [-\frac{1}{2}(\frac{{\partial }^{2}}{\partial {x}^{2}}+\frac{{\partial }^{2}}{\partial {y}^{2}})+{\mu }_{2D}({n}_{2D},a)\\  &  & +\frac{1}{2}({\gamma }^{2}{x}^{2}+{\nu }^{2}{y}^{2})]\,{\varphi }_{2D}(x,y,t),\end{array}$$

with the normalization $\int \,dxdy|{\varphi }_{2D}(x,y,t){|}^{2}=1$, where

13 $${\mu }_{2D}({n}_{2D},a)=\frac{4\pi \frac{\sqrt{{n}_{2D}}}{\sqrt{2\pi }{d}_{z}}({x}_{2D}+\frac{2\alpha {x}_{2D}^{2}\sqrt{a}}{\sqrt{5{d}_{z}}{\pi }^{1/4}})}{1+\frac{\alpha {x}_{2D}\sqrt{a}}{\sqrt{5{d}_{z}}{\pi }^{1/4}}+\frac{8\alpha {\pi }^{7/12}}{\sqrt{6}\eta }{(\frac{a{x}_{2D}^{2}}{{d}_{z}})}^{5/6}},$$

with ${d}_{z}=l/\sqrt{\lambda }$. Similarly, if we have a stronger trap in *x*, *y* directions ($\gamma ,\nu \gg \lambda$), the quasi-1D nonlinear Schrödinger equation for the crossover is given by (details in Methods):

14 $$i\frac{\partial {\varphi }_{1D}(z,t)}{\partial t}=[\,-\,\frac{1}{2}\frac{{\partial }^{2}}{\partial {z}^{2}}+{\mu }_{1D}({n}_{1D},a)+\frac{{\lambda }^{2}{z}^{2}}{2}]\,{\varphi }_{1D}(z,t),$$

with the normalization $\int \,dz|{\varphi }_{1D}(z,t){|}^{2}=1$, where

15 $${\mu }_{1D}({n}_{1D},a)=\frac{\frac{2}{{d}_{x}{d}_{y}}({x}_{1D}+\frac{4\alpha }{5\sqrt{\pi {d}_{x}{d}_{y}}}a{x}_{1D}^{3/2})}{1+\frac{2\alpha }{5\sqrt{\pi {d}_{x}{d}_{y}}}a{x}_{1D}^{1/2}+\frac{8\alpha {\pi }^{1/6}}{3\eta {({d}_{x}{d}_{y})}^{5/6}}{a}^{5/3}{x}_{1D}^{5/6}},$$

with $${d}_{x}=l/\sqrt{\gamma },\,{d}_{y}=l/\sqrt{\nu }$$.

### Numerical Results

The 3D, quasi-2D, and quasi-1D crossover Eqs (10), (12) and (14) do not have analytic solution and different numerical methods, such as split-step Crank-Nicolson^43,44^ and Fourier spectral^70^ methods, can be used for their solution. In the following we undertake the study of vortex-lattice formation from a solution of the relevant equations by the Crank-Nicolson method^45^.

In particular, we compare the results from the crossover model, given by the nonlinear Schrödinger equation (11) with *μ*(*n*, *a*) obtained from Eq. (4), with the Gross-Pitaevskii equation, that is Eq. (11) with Eq. (5). For this study we take a trapped disk-shaped Bose gas of 500 atoms in the *x*-*y* plane with *γ* = *ν* = 1, *λ* = 900. A large *λ* makes a strongly disk-shaped Bose gas to generate stable vortex lattice without transverse instability^54,55^ of the vortex lines. A small *λ*, on the other hand, leads to bent vortex lines^54,55^ which may destroy a clean vortex-lattice structure. Moreover, we take the oscillator length *l* = 1 *μ*m, which for a trapped Bose gas of ^87^Rb atoms corresponds to a trap frequency *ω* ≈ 2*π* × 116 Hz. The scattering length will be taken as tunable to different values near a Feshbach resonance^9,10^.

From Fig. 1 we see that the plots of the universal function *f*(*x*) versus *x* of the Gross-Pitaevskii and the crossover models separate at *x* ≈ 0.4, where beyond mean-field corrections become important and this happens for scattering length *a* > 2000*a*_0_ in this case, where *a*_0_ = 5.2917721067 × 10^11^ m is the Bohr radius. In the strong coupling domain, the results for the observable of a trapped bosonic superfluid as obtained from the Gross-Pitaevskii model and the crossover model will be different. In the case of most observables, such as density, rms sizes, frequencies of oscillation^71^ etc., this difference can be seen only after a careful comparison of the results. However, the vortex-lattice structures of a rapidly rotating trapped bosonic superfluid as obtained from the two models are found to be qualitatively different with widely different number of vortices in the two cases.

**Figure 2:**
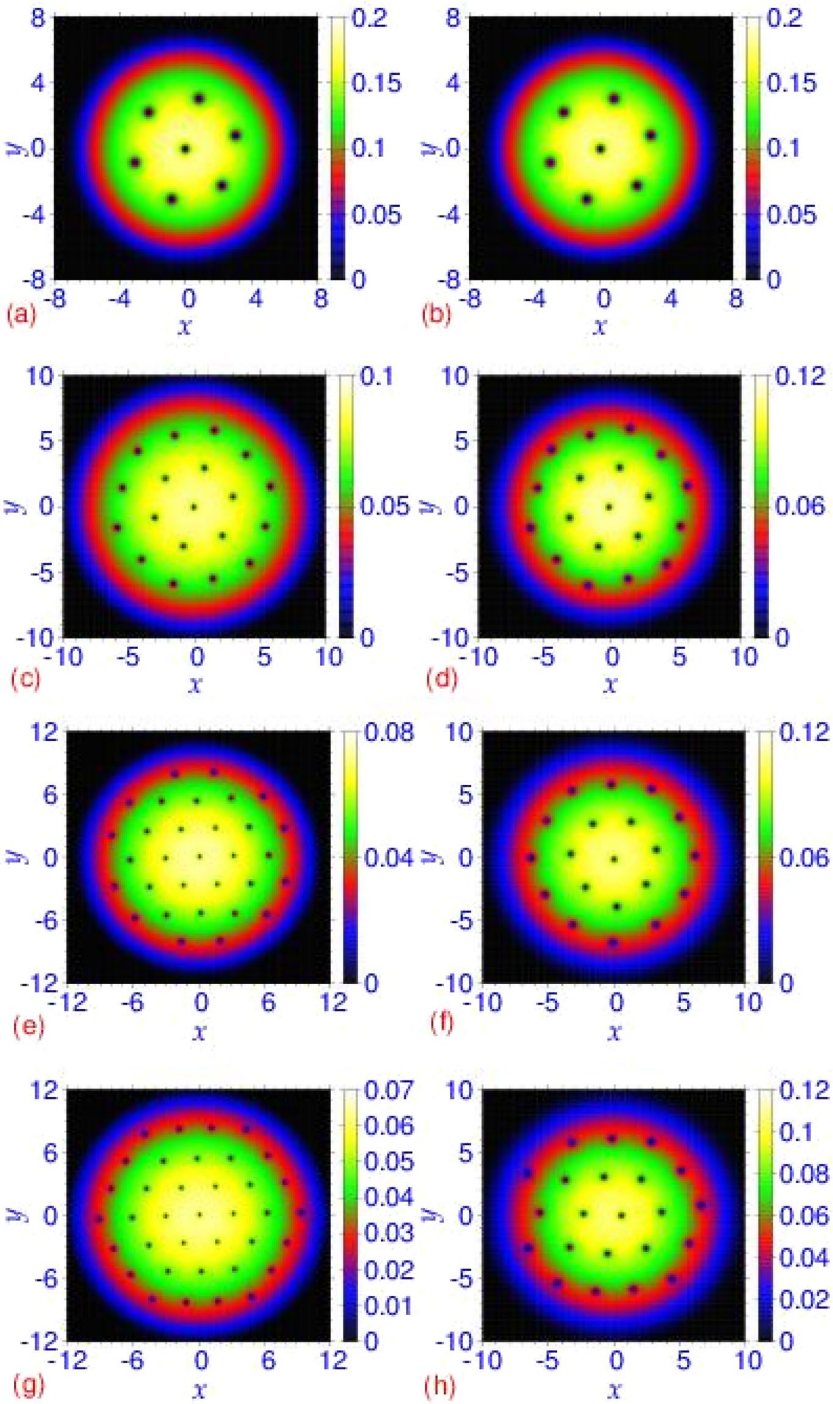
The generated vortex lattice from a contour plot of the reduced 2D density in the *x*-*y* plane *n*_2*D*_(*x*, *y*) from the Gross-Pitaevskii (GP) model, i.e. Eq. (5), and the crossover (CO) model, i.e. Eq. (4), by using the nonlinear Schrödinger equation (11) for (**a**) *a* = 500*a*_0_ (GP), (**b**) *a* = 500*a*_0_ (CO), (**c**) *a* = 2000*a*_0_ (GP), (**d**) *a* = 2000*a*_0_ (CO), (**e**) *a* = 3000*a*_0_ (GP), (**f**) *a* = 3000*a*_0_ (CO), (**g**) *a* = 4000*a*_0_ (GP), (**h**) *a* = 4000*a*_0_ (CO). The other parameters of Eq. (11) are *N* = 500, Ω/*ω* = 0.4, *γ* = *ν* = 1, *λ* = 900, *η* = 4.7, *l* = 1 *μ*m. The plotted quantities in this and following figures are dimensionless.

To illustrate the vortex lattice, we plot in Fig. 2 the reduced 2D density in the *x*-*y* plane

16 $${n}_{2D}(x,y)=\int \,dz|\varphi (x,y,z){|}^{2}$$

of a rapidly rotating trapped bosonic superfluid of 500 atoms with angular frequency Ω = 0.4 obtained from the solution of the Gross-Pitaevskii model and the crossover model for different scattering lengths *a* ranging from *a* = 500*a*_0_ (weak coupling) to *a* = 4000*a*_0_ (strong coupling). In both the Gross-Pitaevskii model and the crossover model, for a fixed number of atoms *N* and a fixed angular frequency Ω, the number of vortices increases as the scattering length increases resulting in a larger *μ*(*n*, *a*): the linear system with *μ*(*n*, *a*) = 0 does not generate vortex lattice independent of the rotational frequency. For *x* > 0.4, the mean-field Gross-Pitaevskii model has larger nonlinearity than the beyond mean-field crossover model, viz. Fig. 1. Hence the Gross-Pitaevskii model generates more vortices than the crossover model in this domain for a fixed angular frequency Ω, viz. Fig. 2(a–h). From Fig. 2 we find that the numbers of generated vortices for *a* = 500*a*_0_, 2000*a*_0_, 3000*a*_0_, and 4000*a*_0_ using the Gross-Pitaevskii model (crossover model) are, respectively, 7 (7), 19 (19), 31 (20), 37 (22), demonstrating a saturation of this number in the crossover model.

**Figure 3:**
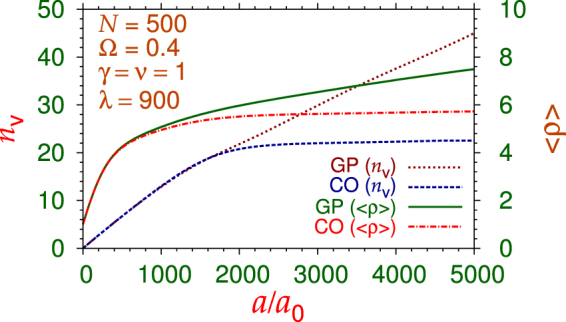
Number of vortices *n*_v_ and the rms size 〈*ρ*〉 in the *x*-*y* plane versus scattering length *a* in a disk-shaped trapped bosonic superfluid of *N* = 500 atoms rotating with angular frequency Ω = 0.4. Results obtained from the nonlinear Schrödinger equation (11) using both the Gross-Pitaevskii model, i.e Eq. (5), and the crossover model, i.e. Eq. (4). The trap anisotropies are *γ* = *ν* = 1, *λ* = 900 and the universal parameter *η* = 4.7.

In Fig. 3 we plot the number of generated vortices *n*_v_ versus scattering length *a* of the Gross-Pitaevskii model and the crossover model. From Fig. 2 we find that the size of the trapped bosonic superfluid in the *x*-*y* plane also increases with the scattering length. To quantify this difference between the two models, we also plot in this figure the rms sizes in the *x*-*y* plane 〈*ρ*〉 of the two models. The number of generated vortices (and the rms size) in the Gross-Pitaevskii model is always larger or equal to that in the crossover model. For smaller values of scattering length in the weak-coupling domain, the nonlinearities of both models are practically the same, and the number of generated vortices (and the rms size) in both models are the same. However, in the Gross-Pitaevskii model the number of vortices (and the rms size) increases indefinitely with the increase of scattering length, whereas the same in the crossover model tends to saturate for *a* > 2000*a*_0_.

**Figure 4:**
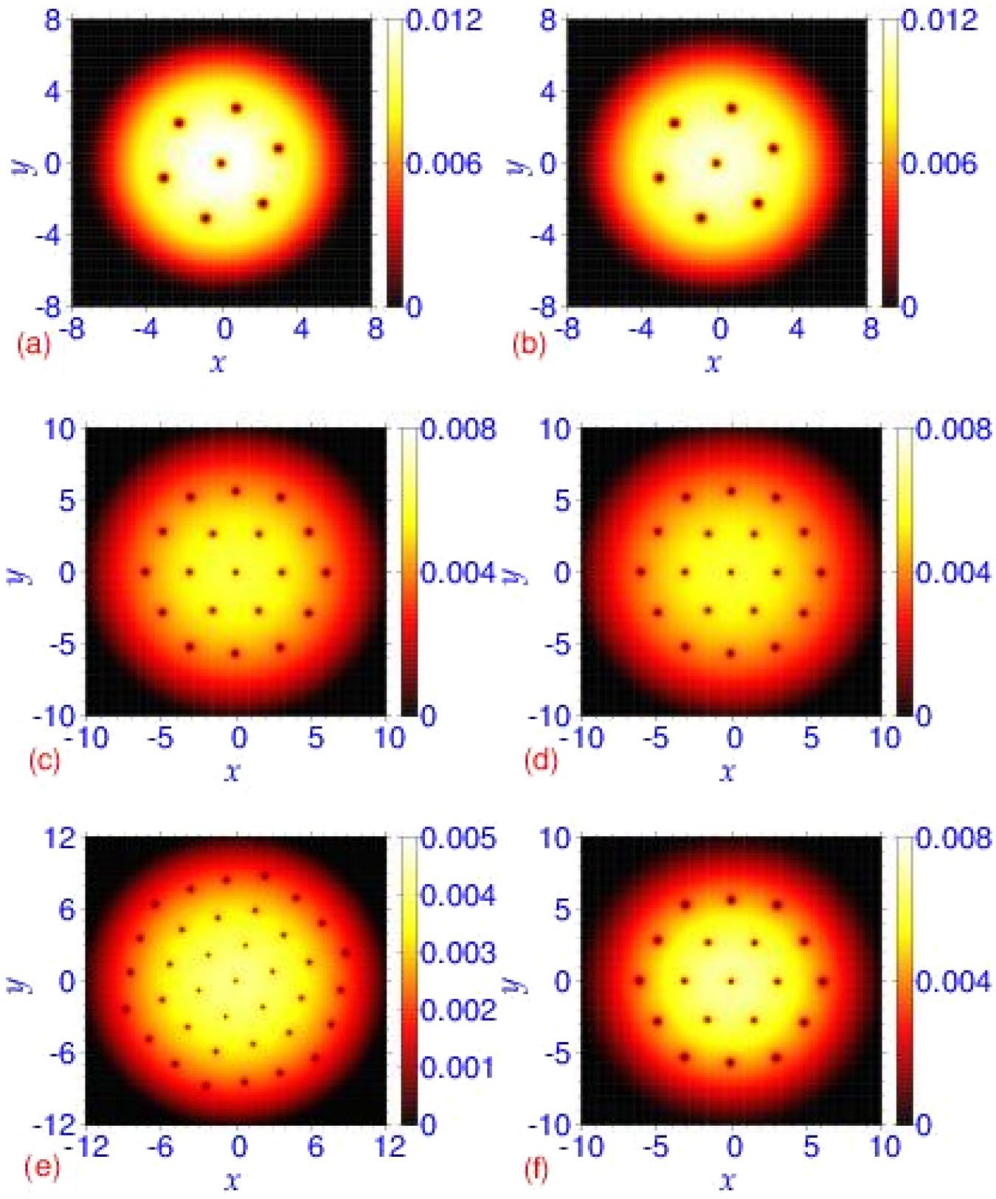
The generated vortex lattice from a contour plot of the 2D density |*ϕ*_2*D*_(*x*, *y*)|^2^ in the *x*-*y* plane from the Gross-Pitaevskii (GP) model, given by Eq. (5) and the crossover (CO) model, given by Eq. (4), by using the nonlinear Schrödinger equation (12) for (**a**) *a* = 500*a*_0_ (GP), (**b**) *a* = 500*a*_0_ (CO), (**c**) *a* = 2000*a*_0_ (GP), (**d**) *a* = 2000*a*_0_ (CO), (**e**) *a* = 4000*a*_0_ (GP), (**f**) *a* = 4000*a*_0_ (CO). The other parameters are the same as in Fig. 2.

Next we use the quasi-2D model (12) to see how well it can describe the vortex-lattice formation in the disk shaped trapped bosonic superfluid studied above using the 3D model (11) while employing the same sets of parameters in 2D and 3D. In Fig. 4 we display the contour plots of the 2D densities for increasing scattering lengths: *a* = 500*a*_0_, 2000*a*_0_, and *a* = 4000*a*_0_ using the quasi-2D Gross-Pitaevskii^72^ and crossover models (31). For the same value of scattering length, the vortex lattice obtained in Fig. 2 using a 3D description is quite similar to that obtained from the quasi-2D model in Fig. 4. For smaller values of scattering length (*a* = 500*a*_0_, 2000*a*_0_), the quasi-2D and the 3D models lead to identical description. For the larger scattering length (*a* = 4000*a*_0_), the number of vortices in the crossover model using 3D and quasi-2D descriptions are 22 and 19, respectively. Considering the large value of the nonlinearity the agreement is quite fair: the quasi-2D model derived by integrating the transverse dynamics is valid for a small nonlinearity.

## Discussion

The commonly used single-orbital Gross-Pitaevskii equation is appropriate to describe a trapped pure Bose-Einstein condensate for a small gas parameter *x*^6^: small values of number of atoms and scattering length. Nevertheless, many experiments deal with a bosonic gases with a larger gas parameter^13–16^, such that the zero-temperature condensate fraction is less than one^5,36^ but the superfluid fraction is still one^5,38^. The Lee-Huang-Yang correction^29^ to the standard Gross-Pitaevskii equation is appropriate to describe such a case. However, the Lee-Huang-Yang correction is not suitable for the strong-coupling unitarity limit for divergent values of the gas parameter. The properties of a bosonic superfluid in the unitarity limit show universal behavior^13–16^ and hence is the topic of many investigations. We presented an analytic expression for the bulk chemical potential of a uniform Bose gas, suitable for the study of a trapped three-dimensional bosonic superfluid along the crossover from weak-coupling to unitarity. This expression has the correct weak-coupling and unitarity limits in addition to the correct beyond mean-field Lee-Huang-Yang correction and faithfully reproduces the results of an accurate microscopic multi-orbital calculation^31^. Adopting this analytic expression along the crossover, we study the evolution of vortex lattice in a rapidly rotating trapped bosonic superfluid along the weak coupling to unitarity crossover by using a 3D single-orbital nonlinear Schrödinger equation, which is equivalent to the generalized hydrodynamic equations of a superfluid at zero temperature. As expected, by increasing the interaction strength at a fixed angular frequency we find dramatic differences in the vortex-lattice structures obtained from the Gross-Pitaevskii model (5) and the crossover model (4). Similar analytic expressions for the bulk chemical potential of quasi-1D and quasi-2D Bose gases are also derived by integrating out the transverse dynamics. We also studied the vortex lattice in the quasi-2D model using the same parameters as the 3D model. For a strong transverse trap, the quasi-2D model provides a good description of the vortex lattice. We find that the number of vortices from the Gross-Pitaevskii and the crossover models could be widely different: that in the Gross-Pitaevskii model diverges in the unitarity limit whereas in our crossover model it remains finite consistent with the underlying physics. Thus, for future experiments with ultracold Bose gases made of alkali-metal atoms in the crossover from weak-coupling to unitarity, our numerical predictions based on the bulk equation of state (5) and the superfluid nonlinear Schrödinger equation (10) can be a quite useful reference.

## Methods

### Quasi-1D configuration

If we have a stronger trap in the *x*, *y* directions ($\lambda \gg \gamma ,\nu$), the dynamics in these directions can be frozen to be confined in the harmonic-oscillator ground state of the trapping potential *mω*^2^(*γ*^2^*x*^2^ + *ν*^2^*y*^2^)/2:

17 $${\rm{\Phi }}(x,y)={(\pi {d}_{x}^{2})}^{-1/4}{(\pi {d}_{y}^{2})}^{-1/4}{e}^{-{x}^{2}/2{d}_{x}^{2}}{e}^{-{y}^{2}/2{d}_{y}^{2}},$$

where ${d}_{x}=l/\sqrt{\gamma },\,{d}_{y}=l/\sqrt{\nu }$, $l=\sqrt{\hslash /(m\omega )}$ and the wave-function is assumed to have the form^72^

18 $$\varphi ({\bf{r}},t)={\varphi }_{1D}(z,t)\,{\rm{\Phi }}(x,y),$$

where the effective dynamics is assumed to be confined only in the *z* direction. The bulk chemical potential will now have the following 1D form^72^

19 $${\mu }_{1D}({n}_{1D},a)=\int \,\mu (n,a)|{\rm{\Phi }}(x,y){|}^{2}dxdy,$$

where *n*_1*D*_ = *N*|*ϕ*_1*D*_(*z*, *t*)|^2^. Consequently, using Eq. (2) at unitarity, we obtain in the quasi-1D configuration

20 $$\mathop{\mathrm{lim}}\limits_{a\to \infty }\,{\mu }_{1D}({n}_{1D},a)=\frac{3}{5{(\pi \kappa )}^{2/3}}\eta {n}_{1D}^{2},$$

where $\kappa ={d}_{x}{d}_{y}{n}_{1D}^{2}$. The LHY correction (1) in this case becomes

21 $${\mu }_{1D}({n}_{1D},a)=\frac{2{n}_{1D}^{2}}{\kappa }[{x}_{1D}+\frac{2\alpha }{5}\frac{{x}_{1D}^{5/2}}{\sqrt{\pi \kappa }}],$$

where *x*_1*D*_ = *an*_1*D*_. Equations (20) and (21) lead to the following quasi-1D chemical potential valid from weak coupling to unitarity:

22 $${\mu }_{1D}({n}_{1D},a)=\frac{2}{{d}_{x}{d}_{y}}{f}_{1D}({x}_{1D}),$$

23 $$\begin{array}{rcl}{f}_{1D}({x}_{1D}) & = & \frac{{x}_{1D}+\frac{4\alpha }{5}\frac{{x}_{1D}^{5/2}}{\sqrt{\pi \kappa }}}{1+\frac{2\alpha }{5}\frac{{x}_{1D}^{3/2}}{\sqrt{\pi \kappa }}+\frac{8\alpha }{3\kappa \eta }{(\pi \kappa )}^{1/6}{x}_{1D}^{5/2}}\end{array}$$

24 $$\begin{array}{rcl} & = & \frac{{x}_{1D}+\frac{4\alpha }{5\sqrt{\pi {d}_{x}{d}_{y}}}a{x}_{1D}^{3/2}}{1+\frac{2\alpha }{5\sqrt{\pi {d}_{x}{d}_{y}}}a{x}_{1D}^{1/2}+\frac{8\alpha {\pi }^{1/6}}{3\eta {({d}_{x}{d}_{y})}^{5/6}}{a}^{5/3}{x}_{1D}^{5/6}},\end{array}$$

where *f*_1*D*_(*x*_1*D*_) is dimensionless. All variables appearing in Eq. (23) are dimensionless, but this expression is not appropriate for a numerical calculation as the denominator therein (*κ*) may have zero. In Eq. (24) this factor in the denominator has been cancelled to yield an expression appropriate for a numerical calculation.

The quasi-1D nonlinear Schrödinger equation is then written as

25 $$\begin{array}{rcl}i\frac{\partial {\varphi }_{1D}(z,t)}{\partial t} & = & [-\frac{1}{2}\frac{{\partial }^{2}}{\partial {z}^{2}}+{\mu }_{1D}({n}_{1D},a)\\  &  & +\frac{1}{2}m{\omega }^{2}{\lambda }^{2}{z}^{2}]\,{\varphi }_{1D}(z,t),\end{array}$$

with the normalization $\int \,dz|{\varphi }_{1D}(z,t){|}^{2}=1$.

For a dilute bosonic superfluid with 3D scattering length *a* and 1D number density *n*_1*D*_, the quasi-1D weak-coupling regime of cubic nonlinearity is obtained if $a/({d}_{x}{d}_{y})\ll {n}_{1D}\ll 1/a$, while the Tonks-Giarardeau regime of quintic nonlinearity of a strictly 1D gas holds if ${n}_{1D}\ll a/({d}_{x}{d}_{y})$^72^. It follows that the quasi-1D strong-coupling Bose gas cannot have the Tonks-Girardeau limit of a strictly 1D Bose gas.

### Quasi-2D configuration

If we have a stronger trap in the *z* direction ($\lambda \gg \gamma ,\nu$), the dynamics in this direction can be frozen to be confined in the harmonic-oscillator ground state of the trapping potential *mω*^2^*λ*^2^*z*^2^/2:

26 $${\rm{\Phi }}(z)={(\pi {d}_{z}^{2})}^{-1/4}\,\exp (\,-\,{z}^{2}/2{d}_{z}^{2}),\,{d}_{z}=l/\sqrt{\lambda },$$

where $l=\sqrt{\hslash /m\omega }$. and the wave-function is assumed to have the form^72^

27 $$\varphi ({\bf{r}},t)={\varphi }_{2D}(x,y,t)\,{\rm{\Phi }}(z),$$

where the dynamics is confined in the *x*-*y* plane. The bulk chemical potential will now have the following 2D form

28 $${\mu }_{2D}({n}_{2D},a)=\int \,\mu (n,a)|{\rm{\Phi }}(z){|}^{2}dz,$$

where *n*_2*D*_ = *N*|*ϕ*_2*D*_(*x*, *y*, *t*)|^2^. Consequently, using Eq. (2) at unitarity, we obtain in the quasi-2D configuration

29 $$\mathop{\mathrm{lim}}\limits_{a\to \infty }\,{\mu }_{2D}({n}_{2D},a)=\frac{\sqrt{3}}{\sqrt{5}{\beta }^{1/3}}\eta {n}_{2D},$$

where $\beta =\pi {d}_{z}^{2}{n}_{2D}$. The LHY correction (1) in this case becomes

30 $${\mu }_{2D}({n}_{2D},a)=4\pi \frac{{n}_{2D}}{\sqrt{2\beta }}[{x}_{2D}+\frac{\alpha {x}_{2D}^{5/2}}{\sqrt{5}{\beta }^{1/4}}],$$

where ${x}_{2D}=a\sqrt{{n}_{2D}}$. Equations (29) and (30) yield following quasi-2D chemical potential valid from weak coupling to unitarity:

31 $${\mu }_{2D}({n}_{2D},a)=\frac{\sqrt{{n}_{2D}}}{\sqrt{2\pi }{d}_{z}}{f}_{2D}({x}_{2D}),$$

32 $$\begin{array}{rcl}{f}_{2D}({x}_{2D}) & = & 4\pi \frac{{x}_{2D}+\frac{2\alpha {x}_{2D}^{5/2}}{\sqrt{5}{\beta }^{1/4}}}{1+\frac{\alpha {x}_{2D}^{3/2}}{\sqrt{5}{\beta }^{1/4}}+\frac{8\pi \alpha {x}_{2D}^{5/2}}{\sqrt{6}{\beta }^{5/12}\eta }},\end{array}$$

33 $$\begin{array}{rcl} & = & 4\pi \frac{{x}_{2D}+\frac{2\alpha {x}_{2D}^{2}\sqrt{a}}{\sqrt{5{d}_{z}}{\pi }^{1/4}}}{1+\frac{\alpha {x}_{2D}\sqrt{a}}{\sqrt{5{d}_{z}}{\pi }^{1/4}}+\frac{8\alpha {\pi }^{7/12}}{\sqrt{6}\eta }{(\frac{a{x}_{2D}^{2}}{{d}_{z}})}^{5/6}}.\end{array}$$

The quasi-2D Gross-Pitaevskii model corresponds to *f*_2*D*_(*x*_2*D*_) = 4*πx*_2*D*_. The quasi-2D nonlinear Schrödinger equation is then written as Eq. (12), with the normalization $\int \,dxdy|{\varphi }_{2D}(x,y,t){|}^{2}=1$.
